# Integration of ERT and Geotechnical Investigation for River Restoration: A Case Study of Dam Removal Site Characterization

**DOI:** 10.1007/s00267-026-02382-8

**Published:** 2026-01-30

**Authors:** Mohammadyar Rahimi, Clinton M. Wood, Kevin M. Befus, Jordan J. Holt, Graham Thompson, Mersad Fathizadeh

**Affiliations:** 1 Department of Civil Engineering, University of Arkansas, Fayetteville, AR USA; 2 Department of Geosciences, Gearhart Hall, Fayetteville, AR USA; 3 Watershed Conservation Resource Center, Fayetteville, AR USA

**Keywords:** River Restoration, ERT, Subsurface Characterization, Dam Removal, Fluvial Geomorphology

## Abstract

This study presents a comprehensive subsurface characterization methodology integrating electrical resistivity tomography (ERT) geophysics with traditional geotechnical investigations for river restoration planning at a former dam site. The investigation was conducted on Little Sugar Creek (contributing watershed area of 222 km² and mean annual discharge of 7 m³/s) in Bentonville, Arkansas, following the 2021 failure of the 5-m tall Lake Bella Vista dam. Eleven ERT surveys, incorporating both terrestrial and underwater electrodes, were validated with thirteen borehole investigations to characterize subsurface conditions across the study area. Results showed that near-surface low-resistivity zones (<100 Ωm) corresponded to water-saturated fine-grained sediments are prone to erosion, while moderate-resistivity layers (100–600 Ωm) indicated more stable coarse-grained materials. High-resistivity zones (>1000 Ωm) marked competent bedrock. The findings indicate that the creek is currently undergoing distinct geomorphic adjustment phases in different reaches after dam failure, characterized by the accumulation of coarse-grained deposits such as gravel bars and increased susceptibility to erosion in cases of severe flooding. The “event-driven” phase of adjustment is particularly erosive in the upstream area, where thick sediment deposits and shallow shale bedrock provide less resistance to erosion. The integrated approach enabled detailed mapping of bedrock topography, identification of erosion-susceptible areas, and characterization of subsurface material distribution, providing valuable information for restoration design. This methodology demonstrates the value of combining geophysical and geotechnical techniques for comprehensive subsurface characterization in river restoration projects. Additionally, the approach outlined in this study can be adapted to other river systems undergoing geomorphic adjustments, particularly in post-dam removal environments, to better inform restoration and erosion mitigation strategies.

## Introduction

Dam removal is a common river management strategy, aimed at mitigating geomorphic and ecological degradation from aging or obsolete infrastructure (Chu and You 2021). With many small dams facing an increased risk of failure during high-flow events due to structural deterioration, removal is also driven by safety concerns, loss of functionality, or economic factors. At the reach scale, dam removal triggers a sudden and significant drop in the local base level, initiating channel incision within the former reservoir and potentially upstream (Doyle et al. 2002; Mansfield et al. 2024). The exposed bed and bank morphology -defined by sediment type, grain size, stratigraphy, and bedrock depth- governs the river’s subsequent evolution under hydrodynamic forces.

Channel recovery following dam removal progresses in two distinct phases that are influenced by sediment characteristics and flow regimes (Pizzuto 2002; Fields et al. 2025). The initial “process-driven” adjustment phase involves rapid channel reconfiguration and increased sediment flux, particularly within the former reservoir. As Collins et al. (2024) demonstrated, this phase can erode as much as half of the stored sediments within months, requiring only modest discharges rather than large floods. This rapid adjustment is driven by the increased energy gradient caused by base-level lowering. The process-driven phase is especially pronounced in sand- and mud-dominated impoundments, where finer sediments are transported downstream through bedform migration, suspended-load transport, and other non-discharge-dependent mechanisms such as knickpoint retreat, liquefaction, and mass wasting (Pizzuto 2002; Major et al. 2017). During this process-driven phase, finer sediments are preferentially washed away, leading to bed material coarsening and increased median grain size (Fields et al. 2021).

In contrast, the “event-driven” adjustment phase occurs over a longer period and is predominantly influenced by larger than bankfull flow events. As Collins et al. (2024) explained, this second phase begins once a stable channel forms in the former impoundment that is wide enough to convey the dominant discharge. During this phase, significant erosion needs flood events large enough to overtop the newly established, incised channel banks (i.e., typically flows with recurrence intervals of 5 years or greater). This phase is particularly relevant for gravel-bed rivers, where incision and transport are largely controlled by high-magnitude flow events capable of mobilizing coarse-grained sediments (Pizzuto 2002). As Pizzuto (2002) hypothesized, channel incision in gravel-dominated impoundments often leads to bank instability and eventual collapse, with reworked sediments forming new floodplains and contributing to the development of a quasi-equilibrium channel. This sequence of adjustments can take a decade or more to stabilize without intervention through simple grade control or more comprehensive ecological-based restoration.

The magnitude and timing of these adjustment phases have been documented across multiple dam removal investigations worldwide, revealing how dam characteristics and sediment properties control channel evolution trajectories. The removal of Marmot Dam (14.3 m tall) on Oregon’s Sandy River, which impounded approximately 730,000 m³ of sediment, demonstrated rapid initial adjustments with incision rates reaching 13 m/hr and widening rates of 26 m/hr within 24 hours, followed by sustained evolution as channel width increased from 45 m within two weeks to 80 m after two years (Keith 2012). Approximately 15% of impounded sediment eroded within 60 h, increasing to 50% after one year and 58% after two years. The rapid breaching of Condit Dam (38 m tall) on the White Salmon River, impounding 1.8 million m³ of fine-grained sediment (60% sand, 35% silt and clay), produced extreme responses with 10% of sediment evacuating within 90 minutes and over 60% eroding within 15 weeks (Wilcox et al. 2014). The Elwha River dam removals in Washington resulted in up to 5 m of incision through the Lake Aldwell delta and over 20 m through the Lake Mills delta during the first year, releasing 23% and 37% of reservoir sediments respectively within two years (Randle et al. 2015). Notably, the most extensive lateral erosion occurred in noncohesive deposits during moderate flows exceeding mean annual discharge but below 2-year flood magnitudes. Removal of two dams (36 m and 16 m high) on France’s Sélune River impounding 1.8 million m³ sediments revealed contrasting behavior in lower-energy systems where fine sediment turbidity stabilized within six weeks, yet coarse bed material (D₅₀ of 45–50 mm) remained largely immobile due to low stream power (<30 W/m²) (Fovet et al. 2023). These examples illustrate how interactions among sediment characteristics, removal methodology, valley morphology, and flow regime govern the dominance and duration of rapid versus flood-dependent adjustment phases.

The recovery trajectory of post-dam channels is influenced by site-specific factors such as geological characteristics, sediment stratigraphy, cohesion, depth to bedrock, channel slope, flow regime, valley morphology, sediment particle size distribution, incoming load or upstream supply, and floodplain connectivity (Major et al. 2012; East et al. 2018). These extrinsic controls dictate the overall stability and long-term evolution of the post-removal channel, underscoring the need for robust subsurface characterization to guide river restoration designs.

While both surficial and subsurface geologic conditions influence restoration pathways, traditional sediment sampling techniques often fail to capture the full complexity of riverine deposits. Most stability assessment tools rely on representative bed sediment size distributions to estimate sediment transport rates, evaluate habitat stability, and guide restoration, although the accuracy of these assessments varies significantly depending on the transport methodology employed (e.g., stream power approaches, Meyer-Peter and Muller (MPM), or Yang equations among others) and how well the sampling strategy aligns with the chosen model’s assumptions (Yochum and Reynolds 2018; USACE 2025). Common approaches include surface sampling methods such as bulk point bar material sampling, Wolman riffle pebble counts, direct bed material sampling from the existing channel, and reference reach sampling for restoration planning as well as subsurface investigations such as floodplain borings to assess buried sediments (Shields et al. 2003). However, these point-based methods may not capture the full spatial variability of sediment deposits, particularly in highly dynamic river systems. Due to the heterogeneity of riverine deposits and local geology, even closely spaced borehole drilling may be insufficient to characterize subsurface stratigraphy, sediment thickness, and spatial distribution accurately. Other common pre-restoration investigative methods such as pedological maps and aerial imagery provide some insights into complex river dynamics, but they potentially miss special subsurface conditions that could be essential to understanding river evolution, construction feasibility, and long-term stability (Chambers et al. 2012). Alternative methods such as digital photographic techniques (Purinton and Bookhagen 2019; Garefalakis et al. 2023) and laser particle-size analysis (Miller and Schaetzl 2012) improve grain-size distribution measurements, but they remain spatially limited and cannot resolve deeper subsurface features.

Given these challenges, geophysical techniques offer powerful complementary tools for subsurface investigation in river restoration projects. Among available non-invasive geophysical methods, electrical resistivity tomography (ERT) is particularly well-suited for dynamic riverine environments. Ground penetrating radar (GPR) experiences severe signal attenuation and limited penetration depths in saturated clay-rich sediments, while seismic refraction methods are less sensitive to subtle variations in sediment composition, compaction, and moisture content (Schrott and Sass 2008). In contrast, ERT maintains effectiveness across diverse sediment types and saturation conditions and can be deployed in both terrestrial and submerged configurations for continuous profiling across channel and floodplain environments. Unlike traditional point-based sampling, ERT provides continuous, high-resolution imaging of the subsurface, enabling non-invasive capture of spatial heterogeneity across entire cross-sections. (Chambers et al. 2013; Perri et al. 2014; Rahimi et al. 2024, 2025a). This capability is essential in dynamic fluvial environments where subsurface conditions vary dramatically over short lateral distances due to complex depositional histories, variable flow patterns, and ongoing geomorphic adjustments (Parsekian et al. 2015; Benoit et al. 2019). This spatial continuity, when integrated with direct borehole measurements, overcomes the limitations of point-based methods alone by providing both lateral extent and ground-truth validation of subsurface heterogeneity that is essential for predicting channel evolution and designing effective restoration interventions in post-dam environments. Electrical resistivity variations correlate with sediment compaction, grain size distribution, and moisture content -properties particularly dynamic in post-dam systems undergoing channel incision, bank erosion, and sediment redistribution. By detecting these variations, ERT facilitates a detailed characterization of subsurface conditions that determine erosion susceptibility and resilient restoration interventions. (Doro et al. 2013; Hartvich et al. 2020; Seier et al. 2020; Bellmunt et al. 2022; Rahimi et al. 2025b).

This study integrates 11 ERT survey lines including both terrestrial and submerged electrodes, along with data from 13 boreholes to provide a high-resolution subsurface characterization. This combined approach allows for identifying key stratigraphic boundaries, assessing bank stability, and delineating zones of coarse and fine sediment accumulation, helping to predict channel evolution and guiding river restoration planning. The study site is located on Little Sugar Creek, the site of the former Lake Bella Vista Dam in Bentonville, Arkansas USA that breached in Spring 2021. Post-dam failure restoration efforts were driven not only by the need to address geomorphic changes such as channel incision and bank erosion but also by the desire to restore longitudinal connectivity for aquatic species and to mitigate the loss of aquatic and terrestrial habitats caused by channel instability. While the dam breach accelerated these processes, historical imagery and cross-sectional data indicate that erosion and base level lowering had already been occurring in the upstream reach prior to dam failure. These earlier instabilities were likely influenced by a combination of channel straightening, loss of riparian canopy, upstream land use changes, floodplain infill due to development, and the destabilizing orientation of a nearby bridge. Given the cumulative and ongoing shifts in sediment transport dynamics, a detailed understanding of the subsurface composition can help the design of a restoration approach that accounts for variations in sediment type, stratigraphy, and bedrock depth. This understanding is particularly relevant in the context of post-removal profile alterations and the use of grade control structures, where subsurface conditions influence stability, performance, and long-term success of restoration efforts.

## Study Area

**Fig. 1:**
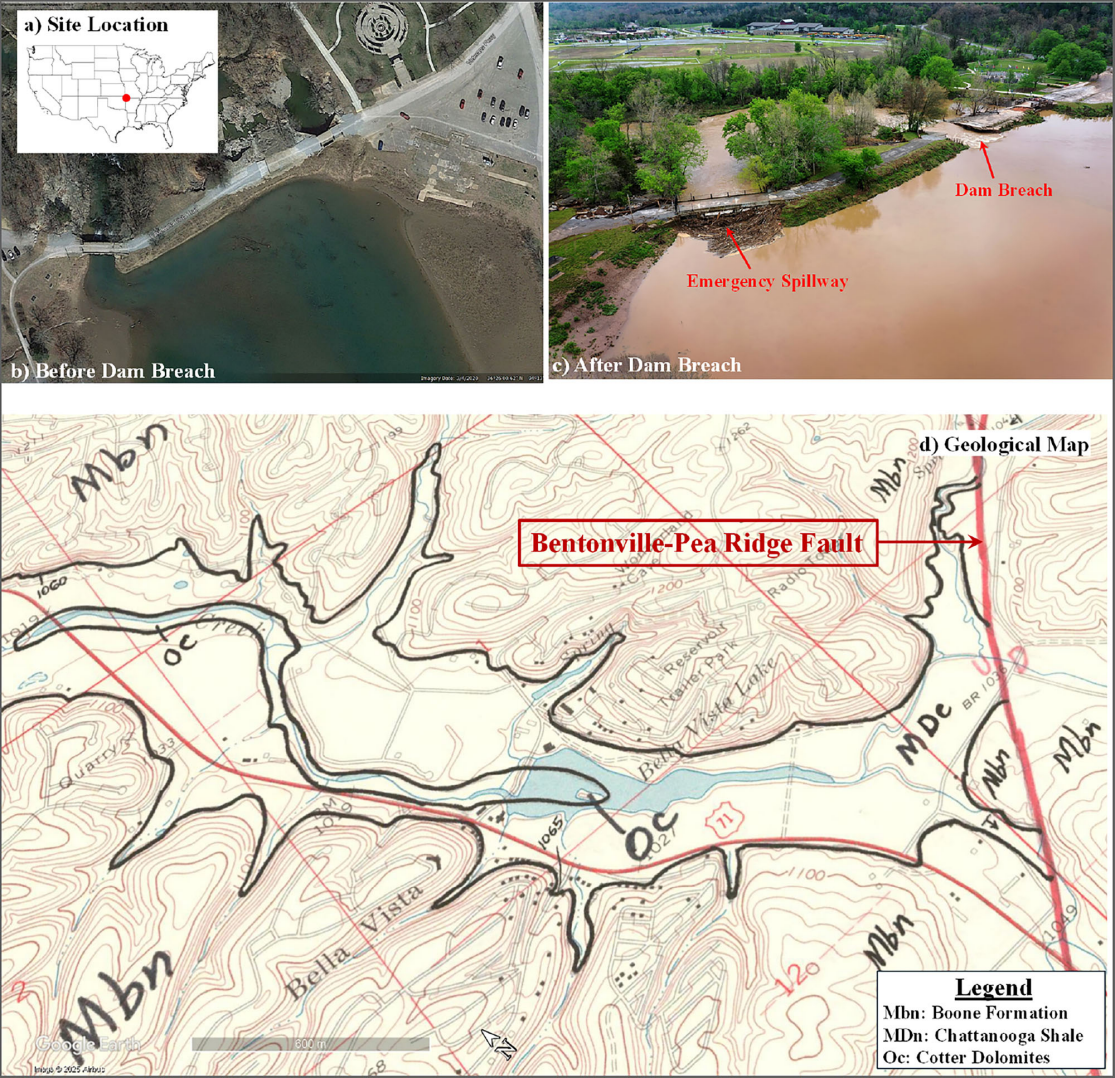
**a** Site location **b** study area before dam breach (from google maps) **c** after dam breach and **d** geological map of the study area showing Boone limestone (Mbn) in the surrounding hills and the Chattanooga shale (MDc) along the study reach with the Cotter Dolomite (Oc) in deeper strata adopted from Glick (1972)

The study site is located at Little Sugar Creek within Bella Vista Lake Park in Bentonville Arkansas (latitude 36.4273°N, longitude 94.2262°W). The project area spans approximately 1.5 km along Little Sugar Creek and its tributary McKisic Creek. The creek bed gradient is about 0.003 m/m, flowing from southeast to northwest. The Lake Bella Vista Dam, about 5 m high structure, was built in 1915, impounded 110,000 m^2^ with a storage volume of 70,300 m^3^ and a contributing watershed area of 222 km^2^. The dam had experienced repeated flood-related damage over the years, including damage by flooding in 2008 and subsequently overtopping by floodwater in 2011, 2013, and 2015, weakening its structural integrity (FEMA 2015). On April 28, 2021, floodwaters breached the dam, washing out part of the structure and scouring the downstream earth below the dam as shown in Fig. 1.

The hydrology of Little Sugar Creek is characterized by seasonal variability in discharge and flood response typical of a runoff-dominated flow regime, where surface runoff from precipitation events drives the majority of streamflow response, creating rapid hydrograph rises and relatively short lag times between precipitation and peak discharge. Cold, perennial, groundwater contribution is common throughout its watershed, typical of karst topography found on the Springfield Plateau, providing important baseflow support during dry periods. At United States Geological Survey (USGS) stream gage 07188838 (Little Sugar Creek near Pineville, Missouri; latitude 36.583889°N, longitude 94.373056°W), located approximately 22 km downstream of the dam, the mean annual discharge over the past 20 years has been approximately 7 m^3^/s, while the mean annual flood is about 328 m^3^/s, with the peak discharge rate of 334 m^3^/s on the day of dam breach. However, there is no USGS stream gage upstream of the dam, making it difficult to directly monitor inflows and hydrologic conditions at the site. These flow conditions highlight the characteristics of runoff-dominated systems in the central US, with the potential for high-magnitude flood events contributing to channel instability and sediment transport.

As shown in Fig. 1d the site is located on the Springfield Plateau, a physiographic province of the Ozark Highlands characterized by shallow, soil-mantled limestone and shale bedrock with karst features and chert interbedding. Surface soils primarily consist of gravelly silt loams formed in well-drained alluvium with limited organic material, more recently influenced by sedimentation from periodic flooding in the failed reservoir. The subsurface geology consists of Quaternary alluvium overlying Mississippian-age Chattanooga Shale formation, which in turn overlies Early Ordovician-age Cotter (or Cotter-Powell) Dolomites as the basement carbonate rock (Haley et al. 1993; McFarland 1998). The alluvial valley is bounded by the Boone Formation, and the Bentonville-Pea Ridge Fault system runs immediately south of the site, potentially influencing local stratigraphic complexity.

**Fig. 2:**
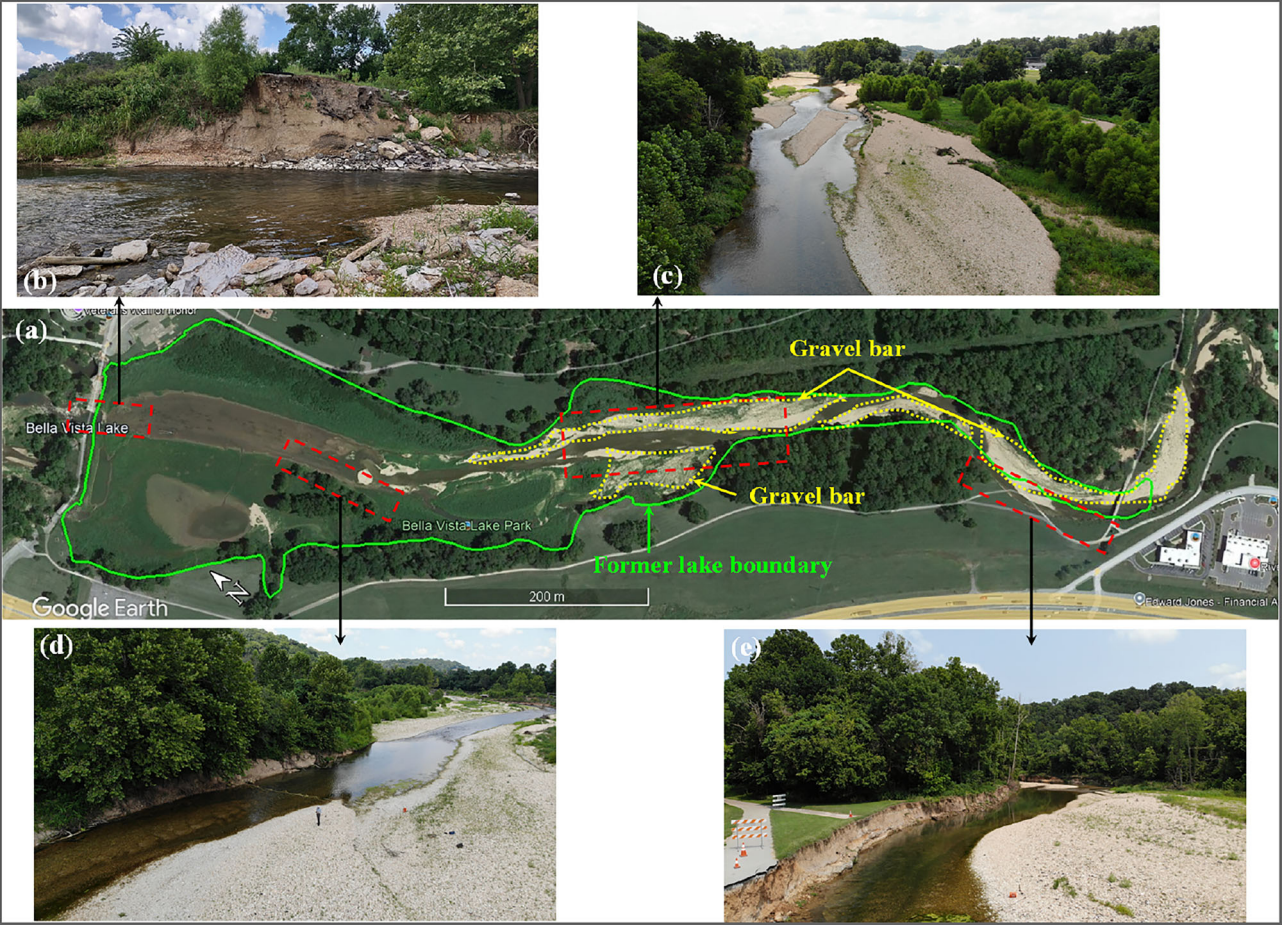
General setting of the study area at the time of testing (**a**) dam location and former lake boundaries, **b** breached section of the dam, **c** gravel bars on the lakebed, **d** bank erosion near the dam, and **e** bank erosion upstream of the lake

The dam failure and subsequent channel adjustments have resulted in significant erosion in multiple areas along Little Sugar Creek, as shown in Fig. 2. The general setting of the study area at the time of testing is provided in Fig. 2a along with the breached section of the dam in Fig. 2b, offering a broad overview of the landscape and key geomorphic features. One of the most severe erosion sites is located upstream of the former lake, where the western bank has eroded significantly, causing a section of a nearby trail to collapse into the creek channel (Fig. 2e). The outcrop at this eroded section reveals a stratified profile, with cohesive soil near the surface and sorted granular deposits below, consisting of loam in the upper strata and gravel in the lower layers. Additional erosion has occurred along the eastern bank, closer to the interior of the former lake. Cross-sectional comparisons from 2017 (pre-failure) to 2023 (post-failure) show significant channel base level lowering in areas with substantial bedrock depth, accelerating streambank erosion processes. Erosion has also developed along the western bank near the former dam axis, where steep slopes indicate significant bank instability (Fig. 2d). This western bank erosion is occurring along what was historically a manmade island, later converted to a peninsula throughout the lake’s operational history, which currently confines floodplain extent. Significant erosion has also developed downstream of the dam on the east bank, where the channel turns abruptly to the west side of the valley, particularly following dam failure. This reach shows the channel deviating significantly from the valley centerline with increasing bedrock depth and changing stream type characteristics, likely reflecting historic channel modifications that pushed the alignment toward the valley wall to increase arable land area.

Beyond bank erosion, gravel bars are deposited across several locations in the former lakebed, often intermixed with silt and sand. An aerial view of these gravel deposits is shown in Fig. 2c, illustrating their spatial distribution. The creek has incised into these deposits, forming a channel approximately 0.5–1.5 m deep. The incision depth increases upstream, likely due to higher stream energy associated with the increased gradient, while it becomes shallower within the former lake where sediment deposition has been more dominant. These ongoing geomorphic changes underscore the need for detailed subsurface characterization to inform restoration planning and stabilization efforts aimed at reducing further erosion and managing sediment transport effectively.

## ERT Methodology

**Fig. 3:**
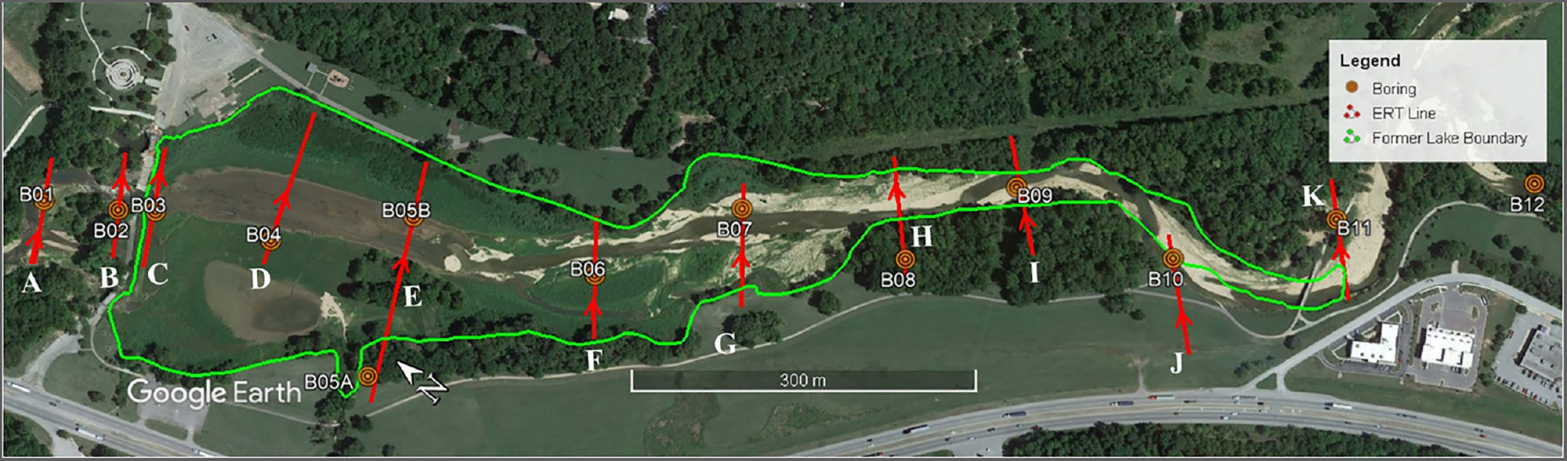
Site map of ERT testing with lines A-K shown along with borehole locations (B01 through B12) and former reservoir boundary. Survey directions are shown by red arrows

The ERT survey at Little Sugar Creek was conducted on July 19, 24 and 25, 2024. The site layout, including ERT line locations, boreholes, and the former reservoir boundary, is shown in Fig. 3. A total of 11 ERT survey lines (labeled A through K in Fig. 3 moving upstream) were deployed. Survey lines ranged from 88 to 222 m in length, incorporating 49-112 electrodes spaced at 2 m intervals. The surveys were oriented from southwest to northeast, generally perpendicular to the valley centerline as indicated by red arrows in Fig. 3. This orientation was chosen to capture historic trends in bedrock erosion, as the channel has moved within the valley over time, and to effectively characterize the stratigraphy of the floodplain and riverbanks across the valley cross-section. This approach helped to avoid the potential loss of valuable subsurface data that could occur by following recent channel positions given the heavy manipulation of the channel during lake establishment. Additionally, continuous straight longitudinal survey lines were not feasible in several locations due to natural barriers including trees, steep riverbanks, and other site-specific obstructions that would have interrupted data continuity.

**Fig. 4:**
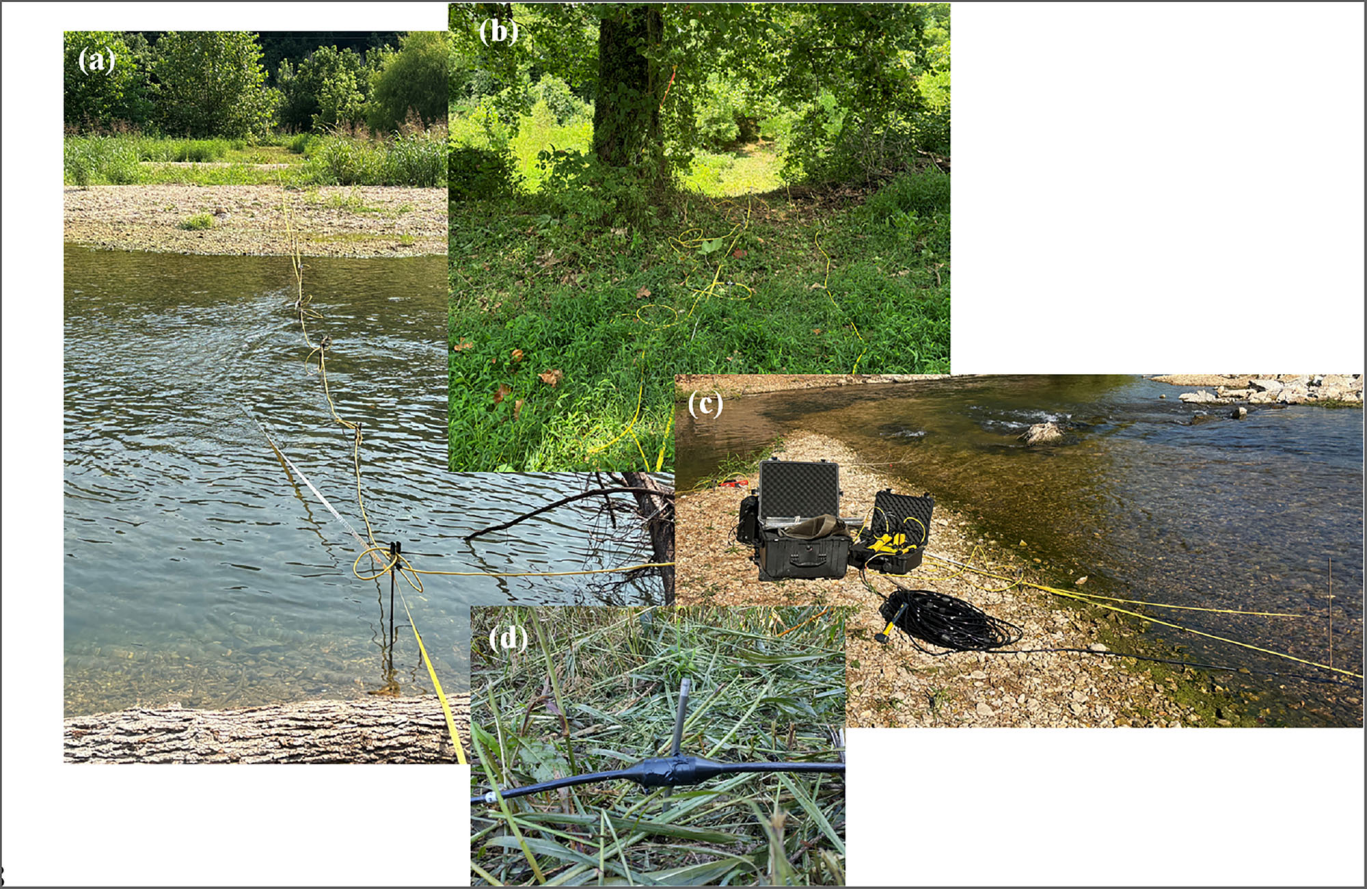
ERT survey setup: **a** ERT line crossing the creek, **b** land-based ERT electrodes, **c** ERT testing equipment with an underwater ERT cable featuring graphite electrodes, and **d** a close-up view of an underwater graphite electrode installed on the land portion of the survey line

Since survey lines crossed the creek, the electrode setup varied depending on water depth. On land, the resistivity cable was attached to stainless-steel stakes, which were driven 15–30 cm into the ground for secure contact. In deeper water segments (greater than 0.3 m), electrodes were installed using underwater graphite electrodes, thus the placement in the water did not require a steel stake. These electrodes were secured on the creek bed to prevent displacement by the current. However, in shallower water sections for ease of installation, longer stainless-steel stakes were simply driven into the streambed and attached to regular terrestrial electrodes instead of graphite electrodes. Figure 4 illustrates the ERT setup, including an ERT line crossing the creek (Fig. 4a). Land-based electrodes are shown in Fig. 4b, while the ERT testing equipment with an underwater graphite electrode cable is shown in Fig. 4c. Figure 4d shows a close-up view of an underwater graphite electrode installed on the land portion of a survey line. At the time of measurement, the ground surface was dry with no recent rainfall. The surveys were conducted during summer low-flow conditions (July) when the water table was relatively stable and stream discharge was minimal, facilitating safe field access and consistent electrode contact. It should be noted that resistivity values can vary seasonally due to groundwater fluctuations, temperature effects, and changes in soil moisture content, which may affect the absolute resistivity values but are less likely to significantly alter the relative contrasts between lithologic units that form the basis of stratigraphic interpretation (Chambers et al. 2014). To ensure good electrode-ground contact, terrestrial electrodes were watered before each survey to reduce contact resistance, including areas near gravel bars where dry, coarse-grained materials can impede electrical contact. Contact resistance was measured prior to data acquisition and was generally below 2000 Ω, although higher values (up to approximately 3000–4000 Ω) were occasionally observed in areas near gravel bars despite watering. Submerged electrodes maintained consistently low contact resistance due to direct immersion in creek water and did not require additional treatment. River water resistivity was measured in-situ at 27 Ωm, which was incorporated into the inversion process as a boundary condition over the submerged portion of the survey. No additional filtering or calibration was applied specifically to account for contact resistance variations, as the measured values remained within acceptable ranges for reliable data acquisition (generally <5000 Ω). Electrode elevations obtained from Real-Time Kinematic (RTK) GPS were also included in the inversions.

Two-dimensional dipole-dipole and strong gradient survey configurations were selected for the surveys due to their efficiency with multichannel devices, short acquisition times, lack of need for a remote electrode, and ability to provide high lateral resolution (White et al. 2023). Raw ERT datasets were processed using AGI’s EarthImager2D software (Advanced Geosciences 2014). The inversion process aimed to minimize the misfit between measured and modeled resistivity data. To improve inversion stability, individual data points with 20–50% misfit at the end of the inversion run were removed in subsequent iterations. In this study, approximately up to 15% of the data was eliminated based on this criterion to ensure reliable results. The inversion quality was evaluated using the root mean square (RMS) misfit, which represents the average discrepancy between measured and modeled resistivity values. After multiple iterations, a final RMS misfit of about 5% was achieved for each profile. While ERT provides detailed subsurface imaging, it is important to acknowledge the inherent non-uniqueness of resistivity inversion meaning multiple resistivity models can fit the observed data equally well (Loke et al. 2013). Therefore, geophysical results were integrated with borehole data to enhance interpretation reliability and geological accuracy. Table 1 presents characteristics of each survey line including number of electrodes, line length, number of trials, percent data removed, number of iterations and associated RMS error.

**Table 1:** Characteristics of each ERT survey and inversion

ERT Line	Number of Electrodes	Line Length (m)	Number of Trials	Data Removed (%)	RMS Error (%)	Number of Iterations
**A**	51	100	3	6.2	4.98	4
**B**	49	96	1	–	4.86	5
**C**	56	110	2	10.1	4.49	4
**D**	72	142	3	15.7	5.07	5
**E**	112	222	2	5.1	5.77	5
**F**	56	110	2	4.6	4.44	3
**G**	56	110	1	–	4.55	4
**H**	56	110	2	5	4.08	3
**I**	56	110	2	3	5.29	5
**J**	56	110	3	9.6	5.85	5
**K**	56	110	2	4.1	4.5	3

## Borings

Standard Penetration Test borings were drilled on September 4–6, 2024, with designations B01 through B12. Borings were advanced until auger refusal was encountered. From the borings, the uppermost alluvial deposits in the creek bed consisted of poorly graded gravel with sand and silt (GP-GM), clayey gravel with sand (GC), silty sand (SM), clayey sand with gravel (SC) and lean clay with sand (CL) based on Unified Soil Classification System (USCS). The materials varied in density from loose to very dense and exhibited negligible to moderate plasticity. Cohesive lean clay layers (CL) were predominantly found along the banks, indicative of deposition during low-energy flow conditions. Groundwater was encountered at depths ranging from 0.5 to 3.5 m below the surface, with shallower water levels observed in borings closer to the creek. Borings further from the main channel, particularly on terrace surfaces, exhibited lower or absent water levels, indicating variable hydrologic conditions across the site.

**Fig. 5:**
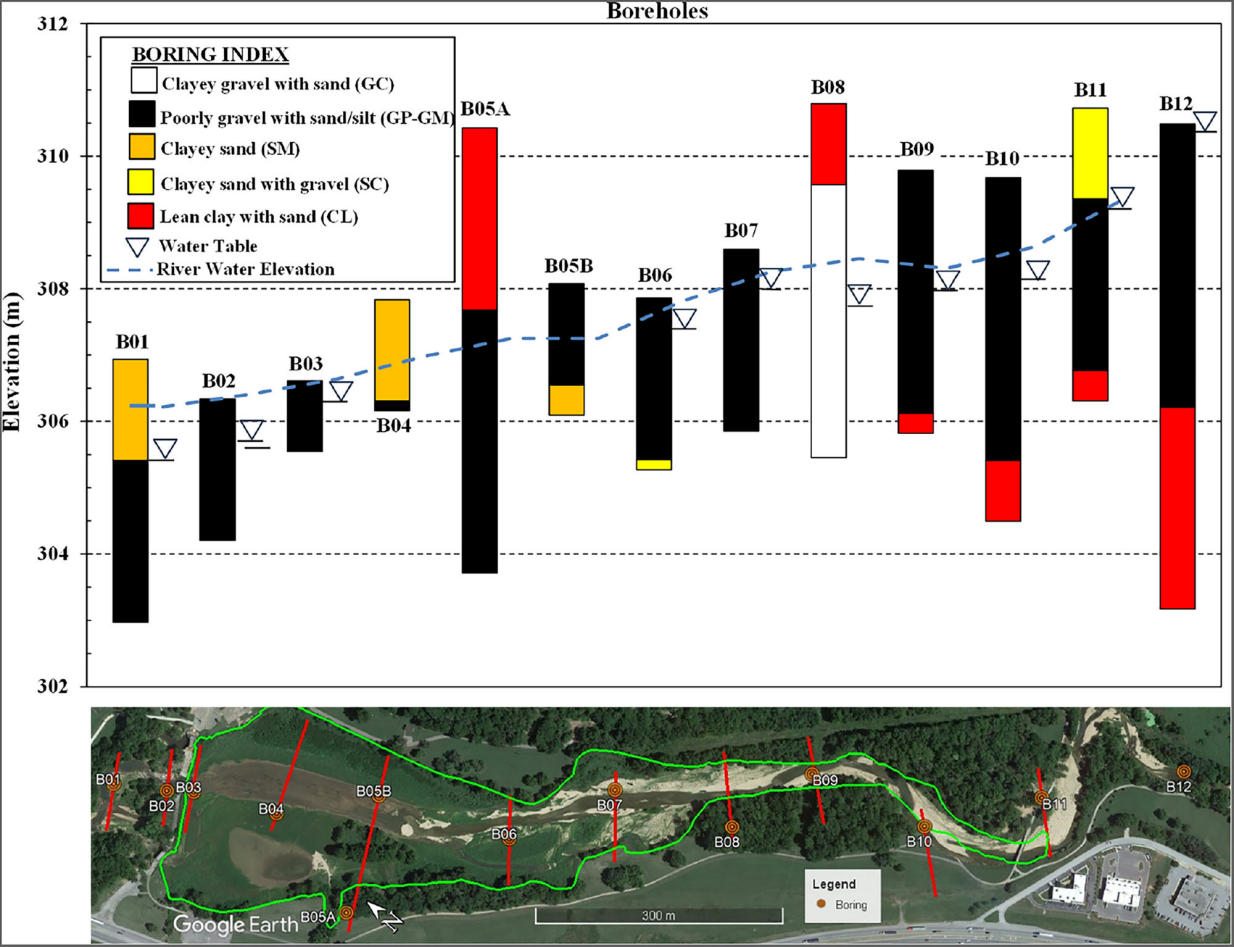
Lithological logs for each borehole along with a map showing approximate locations of boreholes B01-B12. Elevation scales are referenced to the mean sea level (MSL). Blue dashed lines indicate water table elevations at the time of drilling. Borehole logs are arranged from downstream (left) to upstream (right) to facilitate comparison with the ERT profile

The deeper stratigraphy showed significant variability with refusal layers encountered at depths ranging from 0.9 to 7.5 m. Auger refusal is defined as the condition where traditional auger drilling techniques can no longer advance the boring depth. While the determination of refusal is inherently subjective and depends on both equipment capabilities and applied down pressure, refusal was consistently encountered in all SPT borings. Auger refusal typically corresponded to either dense gravel deposits or bedrock. In the southeastern portion of the study area, refusal was predominantly attributed to the weathered Chattanooga Shale formation, encountered between 3.5 and 7.5 m depth. This formation exhibited a moderately hard to hard consistency with low to moderate plasticity. In the northwestern portion of the site, auger refusal was frequently encountered in carbonate bedrock, presumed to be Cotter Dolomite based on regional geologic mapping, although Clifty Limestone could be an alternative interpretation. From an ERT interpretation standpoint, the electrical resistivity signatures of limestone and dolomite are essentially identical, and for river restoration purposes, both carbonate rock types exhibit similar erosion-resistant properties and engineering characteristics. This carbonate bedrock formation is known for its erosion-resistant properties and interbedded chert layers (McFarland 1998). Some borings, particularly those within the boundary of the former reservoir, encountered dense gravel deposits above the bedrock surface, resulting in refusal before reaching the underlying carbonate bedrock. Figure 5 shows the lithologic logs from all boreholes along with their spatial distribution across the site, illustrating the vertical and lateral variability in subsurface conditions that informed ERT interpretation.

## Results

The ERT measurements are detailed in this section. Each 2D line and cross section is referenced to mean sea level (MSL) using GPS measurements, with electrode locations marked by solid rectangles on the profiles. The ERT survey results are organized into three distinct areas: the location of the former dam and downstream area (Lines A-C), survey lines within the former dam reservoir (Lines D-H), and survey lines upstream of the former dam reservoir (Lines I-K). Borehole data were not incorporated as constraints during the ERT inversion process; instead, inversions were performed independently using standard geophysical processing procedures. Boreholes drilled along the survey lines were subsequently utilized to validate the ERT results through post-inversion comparison, providing ground-truth validation of the geophysical imaging and enabling development of site-specific resistivity ranges for different lithologic units.

The interpretation of ERT results requires understanding how material properties influence resistivity values. The primary controlling factors include grain size distribution, water content, porosity, clay content and compaction state (Abidin et al. 2017; Lévesque et al. 2023). These properties exhibit significant variability in alluvial river environments, and their combined effects can complicate the interpretation of resistivity results. It is important to note that ERT interpretation is inherently site-specific, as local geology, hydrogeology, and material properties can significantly influence the resistivity ranges associated with different materials. For this study area, site-specific resistivity ranges were established through post-inversion comparison of ERT profiles with borehole lithology at intersecting locations. This validation process involved correlating observed resistivity values at borehole positions with logged sediment types and refusal depths to develop empirical resistivity-lithology relationships. Based on the integration of borehole data and ERT measurements, the following site-specific resistivity ranges are established: fine-grained and saturated materials typically show resistivity values less than 100 Ωm, while gravel and weathered rock exhibit resistivity between 100 and 600 Ωm. The transition zone spanning the weathered rock to bedrock is characterized by resistivity values between 600 and 1000 Ωm, and more competent carbonate bedrock shows resistivity values exceeding 1000 Ωm. These ranges serve as guidelines for interpretation within our study area, although the precise delineation of subsurface conditions may vary across the site.

### Near the Former Dam Body

**Fig. 6:**
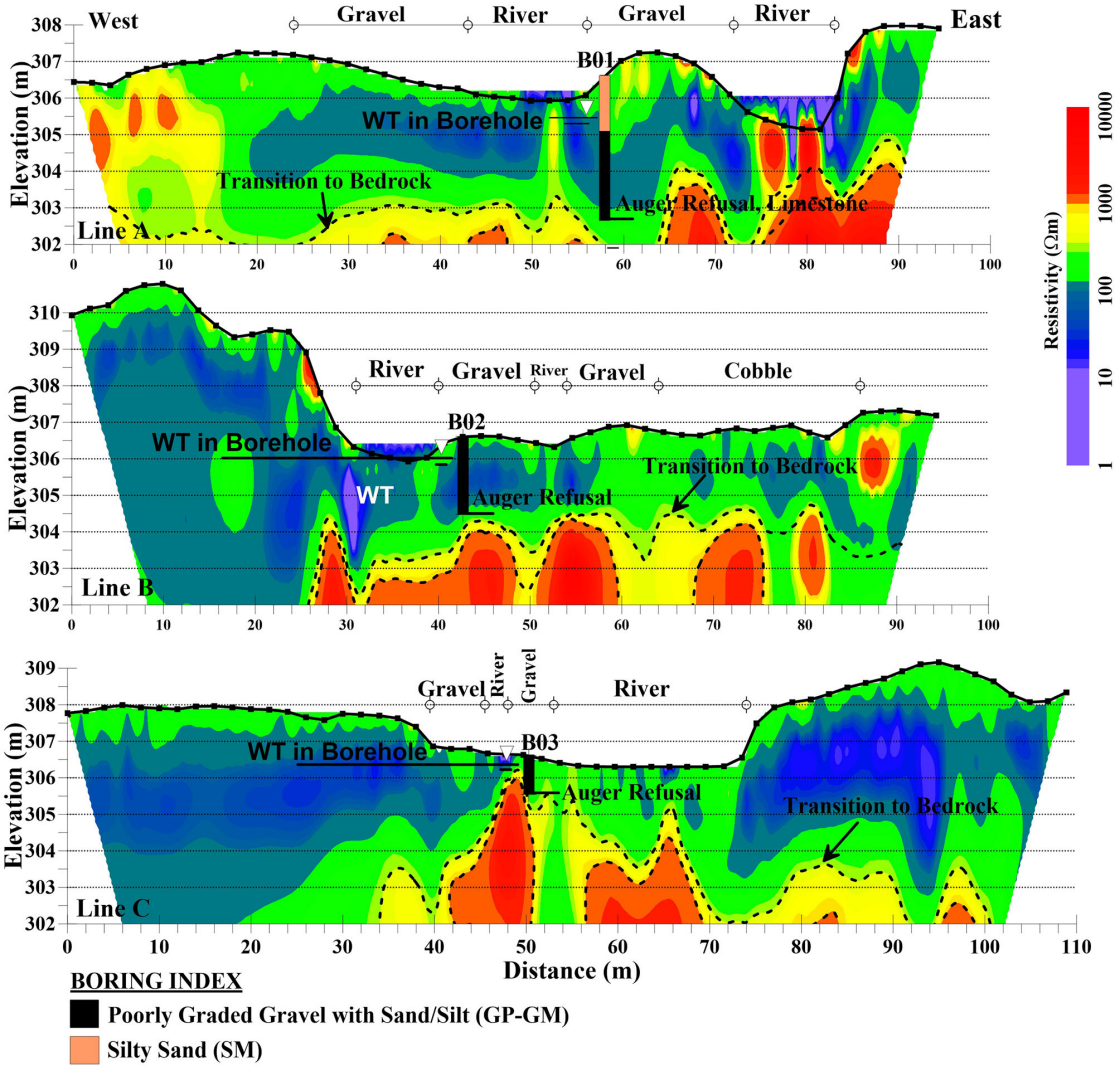
ERT lines near the former dam with intrusive boring logs. Transition to bedrock specified with contours of 600 Ωm and 10,000 Ωm (dashed lines) are shown in the figure. Solid black lines mark the groundwater levels encountered in the borings. The points of auger refusal are shown on the profile

The ERT sections A, B and C near the former dam location along with boring logs B01-B03 are shown in Fig. 6. The inverted ERT cross-sections reveal a three-layer structure with distinct resistivity characteristics across all three survey lines. The surface layer (blue/purple colors), exhibiting low resistivity values (<100 Ωm), varies in thickness and distribution. In line A, this layer extends to approximately 3 m depth and is concentrated near the stream in the middle of the section. Here, the surface layer likely consists of sandy materials transitioning into water-saturated gravel, matching the expectation of higher water content in the creek bed. Line B shows a notably thicker low-resistivity layer to the west (distance 0–25 m), which may be associated with remnants of the old dam structure. Line C exhibits similar near-surface low-resistivity zones on both sides of the section, likely representing fine-grained alluvial deposits from the old reservoir, as this line is situated near the upstream toe of the dam. These areas may also indicate highly saturated zones composed of a mix of fine and coarse-grained gravel, particularly on the western side, where a rivulet from the main creek contributes additional water. Similar resistivity patterns have been observed in other river studies, where values below 100 Ωm are typically associated with water-saturated fine-grained sediments (Sass et al. 2008). The presence of saturated fine-grained materials is especially relevant for restoration planning, as they are more prone to erosion during high-flow events or could be used to support riparian wetlands.

Beneath the surface layer and extending laterally is an intermediate layer exhibiting moderate resistivity (green color) with a resistivity range of 100–600 Ωm, likely composed of coarser grained materials and/or partially weathered rock. Its thickness is variable in the lines near the dam body, extending deeper to the west in line A, suggesting a transition zone between surface sediments and underlying bedrock. The moderate resistivity layer is mostly on the east side of the creek in line B, corresponding with observed gravel deposits in the riverbed. For line C, the moderate resistivity zone extends under the stream corresponding to the observed bedrock in the field. The presence of this moderate resistivity layer suggests a vertical transition from alluvial deposits to more consolidated materials. The transition from alluvial deposits to bedrock is marked by changes in resistivity values shown by yellow color with a resistivity contour of 600–1000 Ωm, (shown by dashed lines) indicating a transition from granular material and weathered rock to more competent carbonate bedrock below. Similar resistivity contrasts between alluvial deposits and bedrock have been documented by Chambers et al. (2012) in their study of river valley stratigraphy.

The bedrock layer characterized by the highest resistivity values exceeding 1000 Ωm (brown/red colors), maintains a relatively consistent elevation of 303–304 m across all three profiles with some local variations. These variations could arise from differences in the depth of weathering or historic erosion of the bedrock, as well as potential infilling with river deposits over time. Notable upward-reaching irregularities in the bedrock surface are observed beneath the river, which may be artifacts caused by geometric errors arising from discrepancies between the actual and modeled electrode locations, rather than actual bedrock upcropping. The bedrock is notably less shallow under the former dam location in line C. The bedrock configuration affects post-dam failure channel adjustment, as any potential headcut migration from the low head dam failure has been effectively reduced by the exposed bedrock apparent in ERT line C and field observation. This provides a natural grade control that limits upstream channel incision and helps maintain the stability of the adjustment process.

The three borings (B01–B03) provide ground-truth for the geophysical interpretations. Across all borings, shallow water tables (0.5–1.5 m depth) were encountered, with overlying silty sand (SM) or poorly graded gravel with silt and sand (GP-GM). These materials correlate with low to medium resistivity zones depending on grain size, density, and degree of saturation. Refusal occurred at shallow depths (2–6 m), corresponding to either dense gravel or carbonate bedrock, consistent with the high-resistivity layers. This correlation between grain size, water content, and resistivity values aligns with findings by Chambers et al. (2014) in their study of alluvial deposits. However, resistivity responses did not always align perfectly with soil classification: dual-symbol soils (e.g., GP-GM, SM) and single units with variable saturation showed different resistivity values. This agrees with Lévesque et al. (2023), who emphasized that resistivity is influenced by the mixture of fine- and coarse-grained materials and their density. Intermixed fine-grained, low-resistivity sediments (e.g., clay and silt) and coarse-grained, higher-resistivity materials (e.g., sand and gravel) can result in resistivity values that do not accurately represent the dominant soil type. Lévesque et al. (2023) stated that when a target sediment is mixed with materials exhibiting significant resistivity contrasts, identifying the specific sediment responsible for the measured electrical resistivity becomes challenging without having access to boring data.

### Former Dam Reservoir

**Fig. 7:**
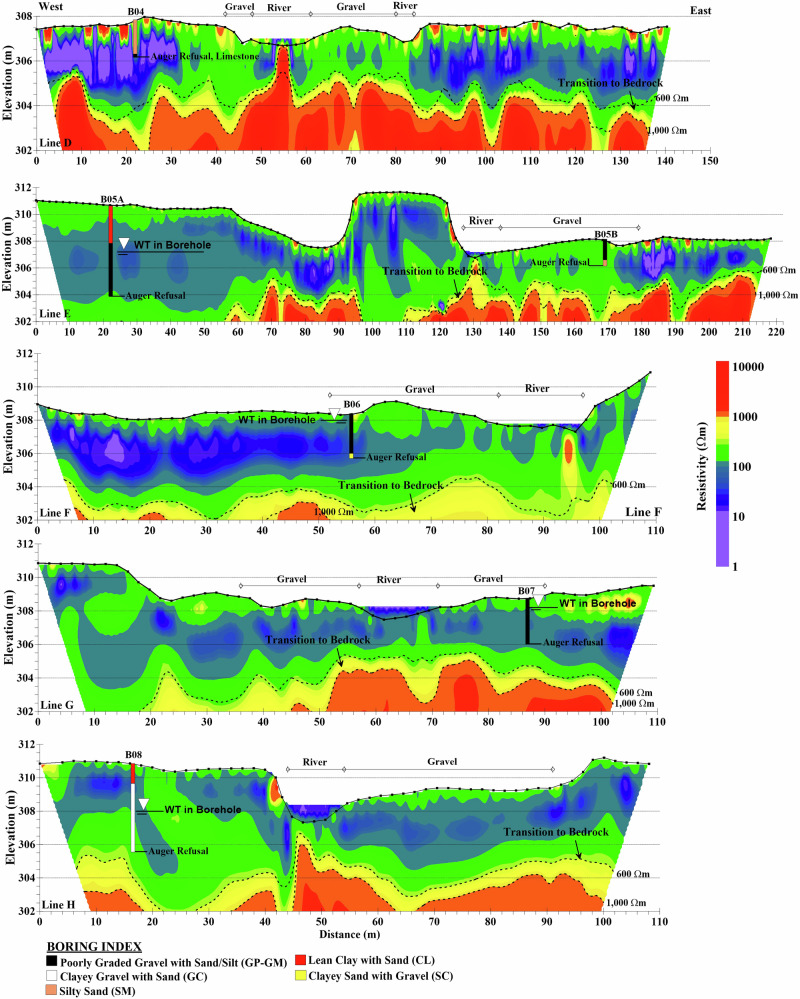
ERT lines in the former dam reservoir with intrusive boring logs. Contours of 600 Ωm and 10,000 Ωm indicate the transition to bedrock. River and gravel bar locations are shown on the profiles. Solid black lines represent groundwater levels encountered in borings and auger refusal points. Note the scale variations across different profiles

The inverted ERT lines D-H in the former dam reservoir along with boring logs B04–B08, creek and gravel bars are shown in Fig. 7. A moderate resistivity layer (100–600 Ωm), underlies the surface deposits across all survey lines, likely representing coarse-grained alluvium or weathered rock consistent with the coarser materials that remained following preferential transport of fines. The moderate resistivity in the central part of line D, for instance, may correspond to gravel deposits, consistent with observations from previous survey lines. Below, there is an intermediate to high resistivity zone (600–1000 Ωm) indicative of transition between surface sediments and bedrock, with its thickness varying laterally across the survey area. The bedrock surface, characterized by high resistivity values (>1000 Ωm), shows some variation in elevation across the survey lines. Line D notably exhibits a slightly shallower bedrock elevation at approximately 303–304 m compared to the other lines (E through H) where bedrock is encountered at around 300–303 m. This variation suggests a gentle dip in the bedrock surface toward the south or local variation in the subsurface geology moving away from the dam body in the upstream area. Shallow gravel deposits near the river channel within the former reservoir exhibit moderate to high resistivity, indicating coarser, well-drained materials that are consistent with the coarser lag deposits remaining after the process-driven sediment sorting. In contrast, deeper gravelly deposits show lower resistivity due to water infiltration. Clayey soils on the river terrace exhibit low resistivity due to high fine content. This pattern aligns with findings from Crook et al. (2008), who reported that transitions between low and moderate resistivity zones often represent boundaries between fine/saturated sediments and coarser, less saturated alluvium.

Borings B04–B08 collectively show a consistent relationship between lithology, water table depth, and resistivity response. Shallow water levels (0.5–3.5 m depth) were encountered across all sites, influencing resistivity more strongly than density contrasts. Fine-grained soils, such as silty sand (SM) and lean clay (CL), are mostly observed on the river terrace, while coarser-grained materials, including poorly graded gravel with silt and sand (GP-GM) or silty/clayey sands (SM/SC), are found primarily in the riverbed. Resistivity often remained low in saturated zones, showing that water content and fines dominate the geophysical response. The ERT section shows low resistivity within saturated sediments, transitioning to medium resistivity at the weathered rock interface by auger refusal, reflecting the combined influence of increasing density, saturation, and lithology on the geophysical response. In summary, fine-grained soils on terraces and saturated coarse gravels in the riverbed generally correspond to low resistivity, while auger refusal at dense gravel or weathered rock coincides with medium resistivity, reaffirming that boring data are essential to interpret subsurface variability in mixed alluvial environments.

**Fig. 8:**
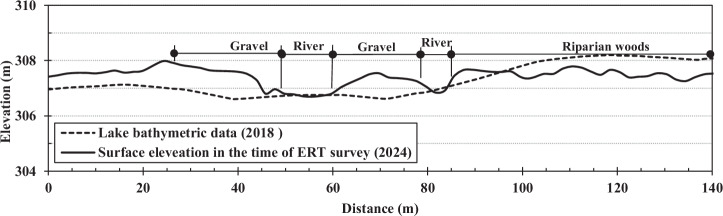
Comparison of the lakebed elevation in 2018 before dam breach and surface elevation after dam breach in 2024 at the time of the ERT survey in the location of ERT line D. Lake bathymetric data (2018) was obtained from a Digital Elevation Model (DEM) using SP80 RTK GPS measurements and 2024 surface data was surveyed with an RTK GPS unit

The ERT results combined with boring data and field observation document clear evidence of the process-driven adjustment phase right after dam failure in this reach. Pre-failure surveys in 2018 documented that the lakebed was largely composed of fine-grained sediments as a surface layer. Following the dam failure, the channel and active floodplains became largely composed of gravel throughout the former lakebed area, indicating preferential transport of finer materials downstream. Cross-sectional comparisons near ERT line D between lakebed elevation before dam breach in 2018 and surface elevation at the time of the ERT survey in 2024 after dam breach (Fig. 8) reveal an increase in elevation of the surface layer to the left and right of the channel, coupled with channel incision. Figure 8 demonstrates general aggradation throughout the lakebed following dam failure, with this settling of material attributed to the sudden widening of the new floodplain within the former lakebed area. This dramatic expansion created a substantial reduction in both depth and velocity during peak storm events, promoting sediment deposition across the wider floodplain surface. Additionally, water backing up from the remnant left and right spillways that remained intact after the partial dam failure further contributed to the reduced flow velocities and enhanced sediment settling conditions. This pattern suggests that the partial dam failure, with remaining left and right spillways, induced setting of coarser materials on the newly formed floodplain while finer sediments were transported downstream. The hydraulic conditions created by this partial failure scenario -characterized by flow expansion, velocity reduction, and backwater effects from the remaining spillway structures- provided conditions for selective deposition of coarser fraction materials within the former lakebed area. However, in areas where riparian woods have established on the riverbanks, current surface elevations remain lower than the original 2018 lakebed elevation, indicating that following the transport of finer sediments, minimal gravel deposition occurred in these vegetated areas due to the stabilizing influence of woody vegetation growth. The contrast between vegetated and unvegetated areas highlights the significant role of vegetation in controlling sediment deposition patterns, with established woody vegetation effectively reducing flow velocities and promoting fine sediment retention rather than coarse material accumulation. The ERT data corroborates this geomorphic evolution, showing the subsurface distribution of materials that resulted from this rapid channel reconfiguration and sediment sorting process.

### Upstream of the Former Dam Reservoir

**Fig. 9:**
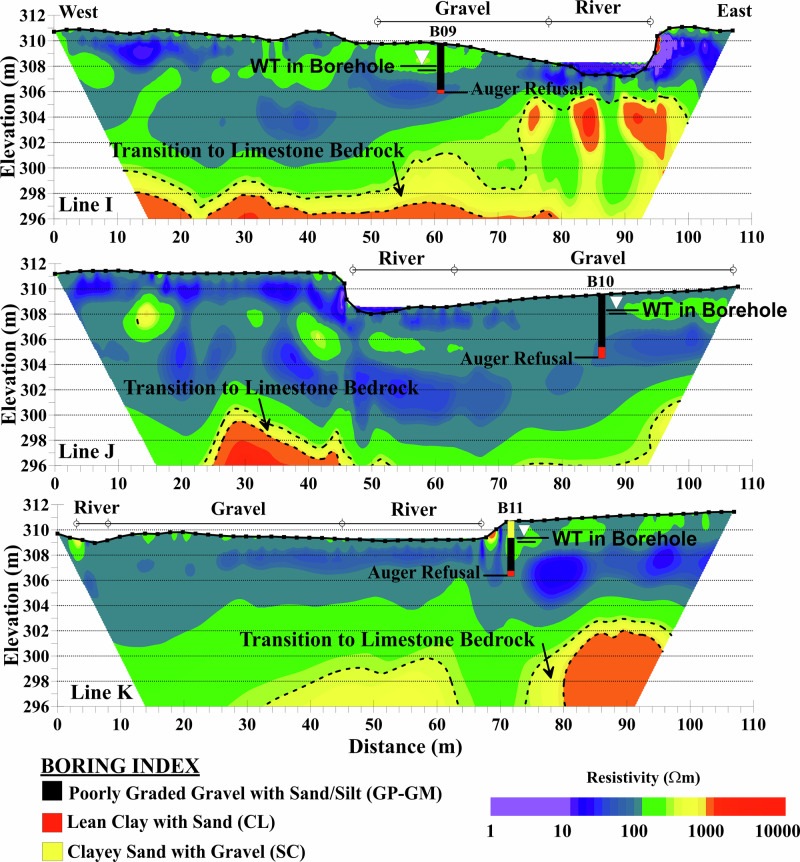
ERT cross sections upstream of the former dam reservoir with boring logs. The locations of the river and gravel deposits are shown on the sections. Solid black lines mark the groundwater levels encountered in the borings and the point of auger refusal

The ERT sections of lines I-K positioned upstream of the former dam reservoir along with the boring logs B09-B11 are shown in Fig. 9. The area has experienced considerable erosion, particularly along the western bank of the river with a higher extent of deposited gravel bars as shown in Fig. 2. The ERT results consistently show low resistivity zones (<100 Ωm) with notable variations in depth and extent. Line I exhibits a near-surface low resistivity zone (0–10 m depth) along most of its length, indicating a thicker accumulation of finer-grained or saturated materials. Lines J and K demonstrate more extensive low resistivity areas, suggesting a greater accumulation of these materials relative to Line I. Notably, all three lines lack near-surface moderate resistivity layers typically associated with gravel bars seen in the creek bed. This absence may be due to a higher fines content mixed with gravel, leading to lower resistivity values than typically expected for pure gravel deposits. As Lévesque et al. (2023) noted, mixed sediments with significant resistivity contrasts require a broader resolved resistivity range to accurately represent their composition. For example, in the case of gravel intermixed with silt and sand, the lower-than-expected resistivity could result from the composite nature of the sediment and the influence of finer-grained materials such as silt, which reduce the overall resistivity.

The remainder of the ERT sections exhibit moderate resistivity (100–600 Ωm) down to an elevation of approximately 300–302 m in different sections. Higher ER areas (~1000 Ωm) are observed east of Line I, potentially indicating variations in material composition or degrees of weathering. The moderate resistivity zones likely correspond to coarser-grained alluvium or shale formations, as documented in boring logs. The carbonate bedrock transition across all three lines occurs at a consistently lower elevation of approximately 296 m compared to previous survey lines (A-H). This transition is marked by an increase in ER, exceeding 1000 Ωm in lines I and J, and above 600 Ωm in line K.

Borings B09-B11 along ERT lines I-K reveal consistent patterns of lithology, water table depth, and resistivity. Fine-grained soils, including clayey sand (SC) and weathered shale (CL), are mostly found on terraces, while coarser-grained materials such as poorly graded gravel with sand (GP-GM) dominate the riverbed. Notably, for all three borings the transition to weathered shale bedrock is not marked by significant resistivity changes, suggesting high saturation levels in the weathered shale with significant resistivity changes only happening in the shale-dolomite transition. The carbonate bedrock transition occurs at lower elevations (~296 m). These observations emphasize that resistivity is primarily controlled by water content and fine-grained material rather than density or lithology changes.

Observations from lines I-K indicate the presence of near-surface shale bedrock rather than carbonate bedrock, as confirmed by boring logs. However, this saturated shale bedrock has not been clearly resolved in the ERT sections. This aligns with findings by Chambers et al. (2012), who reported that saturated clay-rich bedrock exhibited low resistivity values (10–50 Ωm), while overlying valley fill deposits showed much higher resistivity values (200–600 Ωm). A key factor in this challenge is the contrast in weathered zones between shale and dolomite. While both can be clay-rich when weathered, their resistivity responses differ significantly depending on saturation state and degree of weathering. Regional geology indicates the Chattanooga Shale formation typically overlies the deeper Cotter Dolomite in this area. Weathered, saturated shale exhibits low resistivity (similar to fine-grained alluvium) due to its finer-grained composition and higher moisture retention, whereas more competent, less saturated shale shows higher resistivity. Weathered dolomite, although also potentially clay-rich, generally exhibits higher resistivity than saturated shale under similar conditions. This variable resistivity response means that saturated shale can be difficult to distinguish from overlying fine-grained alluvial sediments, and the subsequent shale-to-dolomite transition may also lack a distinct geophysical signature. Consequently, interpretation of shale versus dolomite bedrock in the upstream area relies primarily on borehole lithology and regional geologic stratigraphy rather than ERT signatures alone. The deep bedrock depths in this area further intensify these interpretive challenges. ERT is therefore used to interpolate the lateral extent of stratigraphic units between borehole control points, acknowledging the inherent limitation of resistivity methods in distinguishing between saturated clay-rich materials with similar electrical properties regardless of their degree of consolidation. Future investigations in similar geological settings could benefit from complementary geophysical methods to resolve ambiguous lithologic boundaries where resistivity contrasts are insufficient. Seismic refraction or multichannel analysis of surface waves (MASW) could provide velocity-based constraints that are sensitive to material stiffness and degree of consolidation rather than moisture content, potentially enabling differentiation between unconsolidated alluvium and consolidated shale bedrock (Rahimi et al. 2025a).

**Fig. 10:**
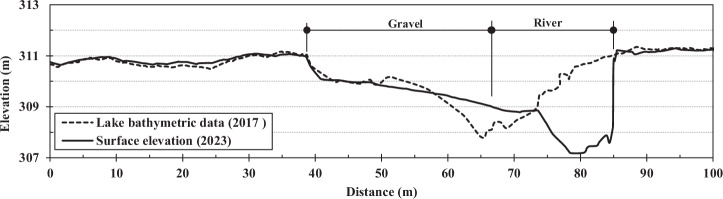
Comparison of the lakebed elevation in 2017 before dam breach and surface elevation after dam breach in 2023 near ERT line I. Lake bathymetric data collected in 2017 was obtained directly using an SP80 Real-Time Kinematic (RTK) GPS. Surface elevation measurements from 2023 were derived from a digital elevation model (DEM), which integrated bathymetric data acquired with the SP80 RTK GPS and topographic data gathered using a DJI Matrice 300 equipped with a Zenmuse LiDAR sensor

Figure 10 illustrates the dramatic channel base level lowering that occurred between 2017 (prior to dam failure) and 2023 (post-failure) near line I, where the significant bedrock depth has contributed to accelerated stream bank erosion, particularly evident on the eastern bank. This temporal comparison demonstrates how the removal of the dam’s base level control has initiated rapid channel incision and lateral erosion processes in areas where deeper bedrock provides less resistance to erosive forces, emphasizing the importance of understanding bedrock depth variations for predicting post-dam removal geomorphic responses.

## Discussion

### Geomorphic Assessment and Restoration Planning

**Fig. 11:**
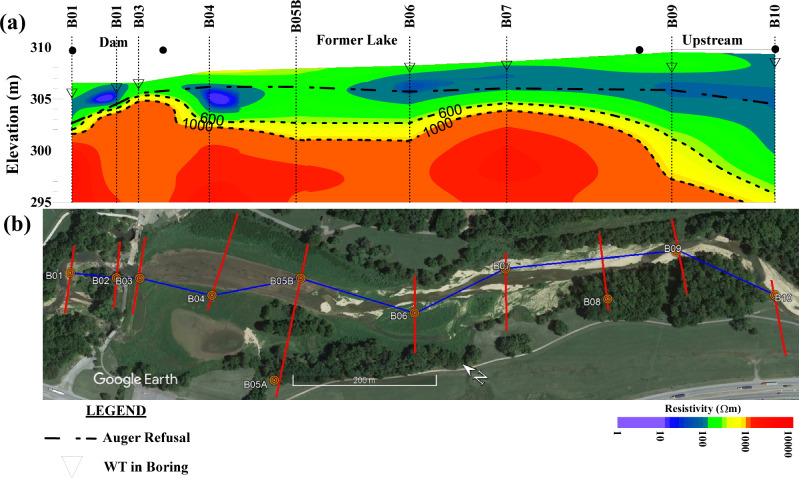
**a** 2D ERT cross section created by interpolating between 1D ERT of profiles extracted from ERT lines A-J at boring locations. **b** Path of the interpolated section on the map. Also, the zones near to the dam, former lake, and upstream areas are shown on the figure

Figure 11 shows a longitudinal ERT section along the creek, constructed to complement the transverse cross-sections and provide an orthogonal view of subsurface variability along the valley axis. This figure is generated by interpolating 1D resistivity profiles extracted from the 2D ERT cross sections of survey lines A-J at each boring location to create a continuous longitudinal section. The horizontal distance between each 1D section along the x-axis in Fig. 11a is the direct distance between the boring locations shown by the blue line in Fig. 11b. While the individual 1D profiles do not provide additional information beyond the transverse 2D sections from which they are extracted, combining them into a longitudinal profile enables visualization of subsurface variations along the river course that cannot be observed in the transverse sections alone. This longitudinal profile is developed to delineate the vertical distribution of sediment and bedrock along the creek’s course.

Figure 11 shows that in the dam vicinity, particularly beneath the dam axis (line C/boring B03), the bedrock is shallowest at approximately 304 m elevation. Bedrock depth increases both downstream and toward the reservoir, indicating either enhanced sedimentation within the former reservoir or increased bedrock erosion downstream. Bedrock depth ranges from approximately 1.5–2 m in the downstream reach to 6–7 m in the upstream area, representing a vertical variability of 4–5 m across the study reach. According to the ERT sections current sediment volume is approximately 220,000 m^3^ with sediment thickness averages 1 m in the downstream zone, 3 m in the former reservoir area, and 5 m in the upstream reach. While auger refusal correlates well with the bedrock transition in the dam area, refusal in the reservoir area typically indicates dense granular material or weathered rock rather than bedrock. This interpretation uncertainty is inherent to both borehole investigations and ERT surveys. Borehole refusal does not always indicate competent bedrock, as dense gravels, cobbles, or weathered rock can prevent auger advancement. Similarly, ERT inversion is subject to non-uniqueness, where different subsurface configurations may produce similar resistivity responses (Everett 2013; Chambers et al. 2012). These uncertainties are addressed by integrating multiple lines of evidence including spatial continuity from ERT, direct sampling from boreholes, and field observations of exposed materials. Where ERT and borehole data agree on bedrock depth and lithology, interpretations are most confident. Where discrepancies exist -such as in the reservoir area- we favor a more conservative interpretation acknowledging that materials may be transitional between weathered bedrock and dense sediment. In the upstream area, the interpreted dolomite bedrock deepens significantly, with refusal depths primarily corresponding to shale bedrock, which lacks a distinct resistivity signature in the ERT section. Water table locations generally align with the upper boundary of low-resistivity zones in the ERT section, except for B03.

The longitudinal profile reveals three relatively distinct morphological zones with implications for river restoration. In the downstream reach, sediment depth progressively decreases with more frequent near-surface bedrock exposures, suggesting a relatively stable channel configuration with limited vertical adjustment potential. The former lake area exhibits a remarkably consistent sediment depth, indicating that the river has likely achieved a state of quasi-equilibrium in this section following dam removal, with balanced sediment transport and deposition processes (Collins et al. 2017). This stabilization pattern resembles observations at Marmot Dam on the Sandy River, where the reservoir reach stabilized after initial rapid adjustments, with channel width reaching 70–80 m within two years and subsequent changes becoming more gradual (Keith 2012), though Little Sugar Creek’s smaller scale likely resulted in less dramatic changes. Similarly, removal of small dams in Wisconsin showed that reservoir reaches stabilized within 1–3 years when sediment volumes were modest and flow energy moderate (Doyle et al. 2005). In contrast, the upstream area demonstrates substantially deeper sediment deposits with a shallow shale bedrock rather than a more stable carbonate bedrock, reflecting ongoing geomorphic adjustment. Channel vertical incision rates in the upstream reach have been estimated at approximately 0.67 m/year based on comparison of cross-sections from 2017 to 2023, while lateral erosion has resulted in bank retreat of about 30 m at some locations over the 6-year post-breach period. The ERT cross-sections reveal that high depth to bedrock and thick loose materials correlate with areas of accelerated erosion and channel incision in the upstream reach and upstream area of the former reservoir. This section, characterized by steeper topographic gradients combined with thicker accumulations of relatively loose sedimentary materials, remains more susceptible to erosion and bank instability. These areas require additional restoration work to achieve stability through floodplain reconnection, including boulder grade control features and greater sinuosity to maintain pool depth.

Erosion susceptibility in different zones is strongly controlled by flow magnitude-frequency relationships. Based on regional streamflow records at United States Geological Survey (USGS) stream gage 07188838, the 2-year flood (Q₂) has an estimated discharge of 276 m³/s, while the 5-year (Q₅) and 10-year (Q₁₀) floods reach approximately 525 m³/s, and 838 m³/s, respectively. The downstream reach, with shallow bedrock and limited sediment cover, is relatively resistant to erosion even during high-magnitude floods. The former reservoir zone should remain stable under flows up to approximately Q₅ but may experience localized bank erosion during larger events. The upstream reach presents greatest concern. Thick, loose sediments over erodible shale bedrock suggest significant geomorphic work occurs during moderate floods (Q₂ to Q₅), with potential for dramatic channel reorganization exceeding Q₁₀. This vulnerability is consistent with event-driven adjustment in gravel-bed rivers following dam removal (Pizzuto, 2002; Collins et al. 2024), where channel evolution is punctuated by floods capable of mobilizing coarse bed material and triggering bank failures.

Climate change projections for the south-central United States indicate increasing frequency and intensity of extreme precipitation events (IPCC 2021), with potential implications for channel stability. Regional models suggest current 10- to 25-year flood magnitudes may occur more frequently under future scenarios. For Little Sugar Creek, this could accelerate upstream erosion where deep, loose sediments over erodible shale create high vulnerability. Even the currently stable former reservoir zone may experience renewed instability if flood frequencies increase sufficiently. These climate risks underscore the importance of designing resilient restoration interventions, including strategic grade control placement and bank protection in areas with deep sediment accumulations over weak substrates identified through ERT. Adaptive management with periodic channel reassessment will be essential for long-term success in a changing climate.

The spatial variations along the longitudinal profile in sediment thickness and composition suggest different evolutionary phases along the river’s course. Approximately four years after the dam breach, the former lake and downstream zones have largely adjusted to post-dam removal conditions, while the upstream area continues active geomorphic adjustment. The presence of significant coarse-grained deposits such as gravel bars, alongside the absence of long-term stabilizing trends, supports the interpretation that the river is currently undergoing an event-driven adjustment where the channel remains prone to further erosion particularly during high-flow events (Doyle et al. 2005; Wilcox et al. 2014). This pattern contrasts with rapid process-driven adjustments observed at Condit Dam on the White Salmon River, where 60% of predominantly fine-grained sediments evacuated within 15 weeks following rapid breaching (Wilcox et al. 2014). Little Sugar Creek’s coarser sediment composition and uncontrolled breach resulted in slower, episodic erosion controlled by flood events. The Sélune River dams in France provide another contrast, fine sediments mobilized rapidly but coarse bed material remained largely immobile due to insufficient stream power (Fovet et al. 2023). Little Sugar Creek occupies an intermediate position, with sufficient energy to mobilize gravel during floods but not the continuous high transport capacity of larger systems like the Elwha River. The upstream erosion pattern also resembles observations from Brownsville Dam removal on the Calapooia River, Oregon, where upstream knickpoint migration and bank instability persisted for several years post-removal in reaches with thick alluvial deposits (Sawaske and Freyberg 2012).

**Fig. 12:**
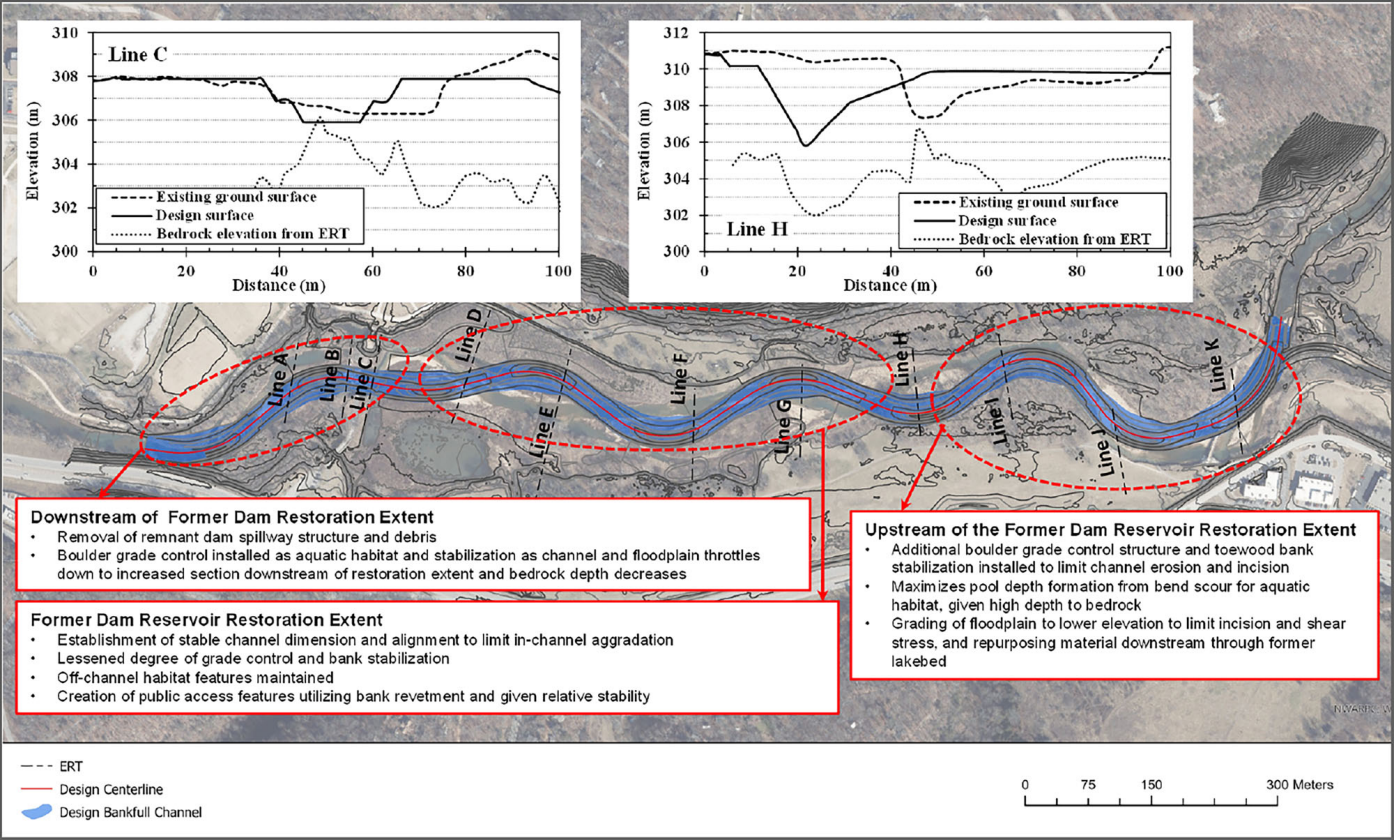
Preliminary restoration plan showing two sample cross-section adjustments. Three different zones of restoration intervention with the relevant restoration efforts are specified on the figure

The current state of the creek as a gravel-bed river suggests that channel incision may lead to progressive bank instability and potential collapse (Collins et al. 2017). As a result, any future restoration efforts should account for the likelihood of continued erosion, particularly during high-flow events, through strategic bank stabilization measures or sediment management strategies to mitigate excessive channel incision and ensure long-term stability. Given that the upstream reach is undergoing event-driven adjustment, restoration interventions in this zone must be designed to withstand high-magnitude episodic flow events while allowing gradual channel adjustment between floods. This requires robust structures and careful construction timing, with work performed during low-flow periods to allow stabilization before the next flood season. A preliminary restoration plan is shown in Fig. 12, incorporating a rerouted river channel and two representative cross-section adjustments. The restoration interventions are designed to stabilize evolutionary processes in different zones based on the distinct geomorphological characteristics identified through ERT analysis. However, this restoration plan is still conceptual, based on subsurface characterization and geomorphic assessment. Final design implementation would require hydraulic modeling to evaluate flow distributions and shear stress patterns under design conditions, as well as sediment transport modeling to assess long-term stability of proposed configurations. The ERT-derived subsurface information provides essential input parameters for such models, including sediment thickness for scour potential assessment, bedrock depth for structure founding, and lateral material property variations for bank stability analysis.

The cross-section modification near ERT line C shows a riffle grading surface designed to work with the existing surface and variable bedrock at or near the surface, where the competent dolomite bedrock provides a natural foundation for channel stability. The shallow, competent dolomite at this location enables direct founding of grade control structures on bedrock, minimizing scour potential and ensuring long-term structural stability. This bedrock-founded approach is preferred in the dolomite reach because it can resist erosive forces during high-flow events without requiring deep excavation or extensive reinforcement. This design allows gravel substrate to form above the bedrock surface with boulders strategically installed for habitat enhancement. The cross-section modification near ERT line H shows a pool grading surface for the restoration, accommodating the variable bedrock below while working with the existing surface. This approach positions the channel closer to coarser-grained materials as indicated by the moderate resistivity area in Fig. 7, with bedrock present on both sides of the channel to enhance stability. The restoration design also increases channel sinuosity both upstream and downstream of line H, near lines I and G respectively, which helps to dissipate stream energy and reduce erosive forces.

These targeted interventions address the specific challenges identified in each morphological zone. In the downstream reach, where bedrock is shallow and the channel is relatively stable, minimal intervention is required. The former lake area, having achieved quasi-equilibrium, may benefit from monitoring and selective bank protection measures. The upstream area, characterized by ongoing geomorphic adjustment and bank instability, requires the most intensive intervention including channel relocation, increased sinuosity, and strategic positioning relative to subsurface materials and bedrock configuration. In the upstream zone where shale bedrock is deeper and less competent than dolomite, grade control structures require different approaches: either deep foundations reaching stable substrates, or flexible designs (e.g., rock vortex weirs, stepped boulder arrays) that accommodate ongoing adjustment. Bank stabilization in this zone requires toe protection keyed into competent materials. Construction timing is another important consideration, as work during or before flood season risks failure before adequate stabilization. The restoration planning incorporates depth of the planned grading surface to bedrock to ensure pool depth is achieved and for keying revetment structures to the hard, less erosive bedrock surface for stability as the native, riparian bank matures to withstand peak flood events. Implementation involves considerations beyond geomorphic characterization, including cost-benefit analysis evaluating investment against infrastructure protection value and ecological benefits, construction feasibility depending on site access and seasonal constraints, and ecological trade-offs where aggressive stabilization may reduce erosion but eliminate habitat features such as undercut banks and woody debris recruitment that supports channel complexity. These multifaceted considerations emphasize that subsurface characterization, while essential, is one component of comprehensive restoration planning integrating engineering, ecological, economic, and social factors.

### Implication for Other Post-Dam Removal Restorations

**Fig. 13:**
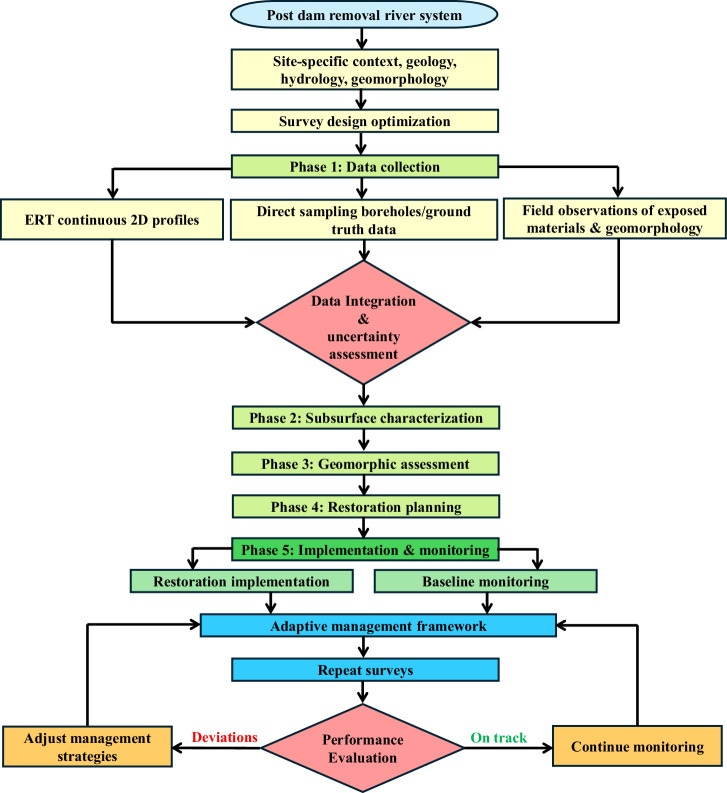
Methodological framework for integrated ERT and borehole investigation in post-dam removal restoration contexts. The framework emphasizes site-specific optimization at the outset (tan boxes at top), followed by five sequential phases (green boxes). The adaptive management loop (bottom portion, blue boxes) illustrates the iterative process where repeat surveys feed into performance evaluation (pink diamond), leading to either adjustment of management strategies or continuation of monitoring (orange boxes)

The integrated ERT and borehole methodology demonstrated in this study provides a general framework for post-dam removal restoration projects, although successful application requires thoughtful adaptation to site-specific conditions as illustrated in the methodological framework (Fig. 13). The framework begins with assessment of site-specific context (geology, hydrology, geomorphology) and survey design optimization before proceeding through five main phases including data collection, subsurface characterization, geomorphic assessment, restoration planning, and implementation with monitoring. While the fundamental principles of combining continuous geophysical imaging with direct ground-truth sampling are broadly applicable, practitioners must calibrate survey design parameters to match local geological, geomorphic, and hydrologic contexts.

Survey design optimization is essential for maximizing data utility while managing project constraints. In this study, the site was divided into three geomorphic reaches (downstream, reservoir, and upstream), each exhibiting distinct sediment characteristics and stability conditions. Other post-dam removal sites may require different spatial divisions depending on valley morphology, sediment distribution, and restoration objectives. Design parameters requiring site-specific optimization include electrode spacing and array configuration (which control depth of investigation and resolution), line orientation relative to valley geometry and anticipated channel evolution patterns, survey line spacing based on expected subsurface heterogeneity, and the balance between terrestrial and submerged electrode deployment. For example, sites with thin sediment veneers over bedrock may require closer electrode spacing to resolve shallow stratigraphic contacts, while deeply incised valleys with thick alluvial fills may prioritize greater investigation depth at the expense of near-surface resolution. Line orientation perpendicular to the valley centerline, as employed here, effectively captures cross-valley stratigraphy and bank conditions, but longitudinal profiles may be more valuable in narrow, confined valleys or where along-stream bedrock variability is the primary concern.

Field implementation methods must also adapt to site-specific physical conditions. In this study, shallow water depth with moderate flow velocity allowed electrodes to be stabilized using long stakes driven into the streambed. However, deeper rivers with slower flow may require different approaches, such as laying electrodes on the bed surface and weighting them with small concrete blocks or sandbags to maintain contact. In very deep water or lake environments, boat-towed electrode arrays or bottom-deployed cables may be necessary. Similarly, the decision to extend survey lines onto floodplains and terraces depends on restoration objectives: if bank stability and floodplain connectivity are primary concerns, extended terrestrial coverage is essential, whereas channel-focused investigations may prioritize denser in-stream coverage.

Geologically diverse settings present varying challenges for ERT applications. At Little Sugar Creek, carbonate bedrock exhibited good resistivity contrast with overlying sediments, enabling clear delineation of the sediment-bedrock interface in the downstream and former reservoir areas. However, in the upstream area, weathered shale bedrock displayed low resistivity contrast with fine-grained alluvial sediments, making it difficult to precisely delineate the boundary between unconsolidated deposits and weathered shale. Similarly, distinguishing between more competent shale bedrock and the underlying carbonate bedrock proved challenging due to insufficient resistivity contrast between these lithologies. In settings with more resistive bedrock (e.g., granite, quartzite) or more conductive sediments (e.g., marine clays, organic-rich deposits), resistivity contrasts may be more pronounced, potentially simplifying interpretation. Conversely, in areas where bedrock and sediment have similar resistivities -such as weathered shale bedrock beneath clayey alluvium- ERT may struggle to resolve the interface, making borehole ground-truthing even more essential. Similarly, highly saline water environments may require different array configurations or processing approaches to account for water column conductivity effects.

Despite these site-specific adaptations, the core methodological principles remain constant: (1) integrate multiple data types to constrain interpretation and assess uncertainty, (2) design surveys to address specific restoration questions rather than applying standardized protocols, (3) calibrate geophysical results with direct sampling at strategic locations, and (4) maintain flexibility to adjust survey parameters based on preliminary results. By following these principles while adapting implementation details to local conditions, the ERT-based approach can inform restoration planning across the diverse range of dam removal contexts, from small run-of-river structures in agricultural landscapes to large hydroelectric facilities in mountainous terrain.

As illustrated in the methodological framework (Fig. 13), successful application begins with careful consideration of site-specific geological, hydrologic, and geomorphic conditions to optimize survey design parameters before data collection. Beyond initial design, ERT offers significant potential for adaptive management through repeat surveys at key intervals or following major floods. Baseline ERT data establishes a reference condition against which future changes can be quantified. Post-restoration surveys could assess whether sediment is accumulating or eroding in design areas, whether scour around structures is developing as predicted, and whether channel migration is constrained as intended. In the upstream reach, where ongoing event-driven adjustment is expected, periodic ERT monitoring could track sediment thickness evolution and identify emerging erosion hotspots before they threaten infrastructure. As shown in the adaptive management loop at the bottom of Fig. 13, this monitoring approach creates an iterative feedback system where repeat surveys inform performance evaluation, which in turn guides decisions to either continue monitoring or adjust management strategies based on observed deviations from expected trajectories. This approach can provide continuous subsurface information across entire survey lines rather than point measurements. Integration of ERT monitoring with cross-section surveys, photo documentation, and habitat assessments would provide a comprehensive adaptive management framework for adjusting strategies if monitoring reveals deviations from expected trajectories. While field implementation methods and survey parameters must be adapted to local conditions -including water depth, flow characteristics, geological setting, and specific restoration objectives- the fundamental integration of continuous geophysical imaging with direct sampling provides a robust and transferable approach for informing post-dam removal restoration planning.

## Conclusion

This study demonstrated the effectiveness of integrating ERT with traditional geotechnical investigations for comprehensive subsurface characterization in river systems. The combined approach enabled mapping of bedrock topology, identification of erosion-susceptible areas, and characterization of subsurface material distribution. Based on the geophysical testing results and boring information thus far, the following conclusions can be drawn:

- The integration of ERT and borehole data identified three distinct resistivity zones corresponding to different subsurface materials: low-resistivity zones (<100 Ωm) indicating erosion-prone saturated fine-grained sediments, moderate-resistivity zones (100–600 Ωm) representing more stable coarse-grained materials, and high-resistivity zones (>1000 Ωm) marking competent bedrock.
- Bedrock elevation varied significantly across the study area, from approximately 304 m near the former dam to ~296 m in the upstream section, which can be used for grade control and channel stability in restoration design.
- Near the dam body and within the former reservoir, carbonate bedrock was detected at varying depths through high resistivity layers in the ERT sections. For upstream lines (I-K), the higher resistivity areas likely indicate the shale/dolomite transition. Borehole logs confirm the presence of shale bedrock below soil deposits in these lines. The transition between soil and shale bedrock could not be accurately identified from the resistivity cross sections since both materials exhibit similar resistivity values.
- The presence of saturated fine-grained materials in low-resistivity zones, particularly in the former reservoir area, indicates areas requiring enhanced erosion protection measures during restoration. These areas are predicted to require toewood revetment and boulder grade control for added protection.
- The findings suggest that the creek is currently undergoing different adjustment phases in different reaches following the dam breach. In particular, there is an “event-driven” adjustment in the former lake and upstream area as evidenced by the accumulation of coarse-grained deposits such as gravel bars. This phase of adjustment increases the likelihood of erosion during severe flood events, particularly in upstream areas where thick sediment deposits and shallow shale bedrock provide less resistance to erosion, which justifies a restoration intervention.
- Borehole logs generally correlate with resistivity profiles across most subsurface layers, although some discrepancies exist at fine-to-coarse material transitions. These variations likely stem from differences in soil saturation rather than stratigraphy alone, as resistivity measurements appear more responsive to water content than grain size.

Overall, this study highlights the importance of understanding spatial variability in subsurface conditions when planning river restoration, delineating horizontal and vertical opportunities and constraints and driving decision-making with respect to profile and planform. The ERT-geotechnical integration directly influenced design decisions by: (1) identifying stable zones with shallow competent bedrock that require minimal intervention, reducing unnecessary costs; (2) targeting erosion-prone areas with deep, loose sediments for intensive protection measures including grade control and bank stabilization; (3) optimizing structure founding depths based on bedrock variability, which shallow bedrock areas enable cost-effective bedrock-founded designs while deep bedrock areas require flexible approaches; and (4) guiding placement of bank protection in zones with erosion-susceptible saturated fine-grained materials. This targeted, zone-specific approach maximizes restoration effectiveness while avoiding intervention in stable reaches.

## Data Availability

Data for the project is available upon request from the corresponding author.
